# Deep Learning Applications in Magnetic Resonance Imaging: Has the Future Become Present?

**DOI:** 10.3390/diagnostics11122181

**Published:** 2021-11-24

**Authors:** Sebastian Gassenmaier, Thomas Küstner, Dominik Nickel, Judith Herrmann, Rüdiger Hoffmann, Haidara Almansour, Saif Afat, Konstantin Nikolaou, Ahmed E. Othman

**Affiliations:** 1 Department of Diagnostic and Interventional Radiology, Eberhard-Karls-University Tuebingen, 72076 Tuebingen, Germany; sebastian.gassenmaier@med.uni-tuebingen.de (S.G.); judith.herrmann@med.uni-tuebingen.de (J.H.); ruediger.hoffmann@med.uni-tuebingen.de (R.H.); haidara.al-mansour@med.uni-tuebingen.de (H.A.); saif.afat@med.uni-tuebingen.de (S.A.); konstantin.nikolaou@med.uni-tuebingen.de (K.N.); 2 Department of Diagnostic and Interventional Radiology, Medical Image and Data Analysis (MIDAS.lab), Eberhard-Karls-University Tuebingen, 72076 Tuebingen, Germany; thomas.kuestner@med.uni-tuebingen.de; 3 MR Applications Predevelopment, Siemens Healthcare GmbH, Allee am Roethelheimpark 2, 91052 Erlangen, Germany; marcel.nickel@siemens-healthineers.com; 4 Department of Neuroradiology, University Medical Center, 55131 Mainz, Germany

**Keywords:** deep learning, DL, MRI, GRE, TSE, prostate MRI, MSK

## Abstract

Deep learning technologies and applications demonstrate one of the most important upcoming developments in radiology. The impact and influence of these technologies on image acquisition and reporting might change daily clinical practice. The aim of this review was to present current deep learning technologies, with a focus on magnetic resonance image reconstruction. The first part of this manuscript concentrates on the basic technical principles that are necessary for deep learning image reconstruction. The second part highlights the translation of these techniques into clinical practice. The third part outlines the different aspects of image reconstruction techniques, and presents a review of the current literature regarding image reconstruction and image post-processing in MRI. The promising results of the most recent studies indicate that deep learning will be a major player in radiology in the upcoming years. Apart from decision and diagnosis support, the major advantages of deep learning magnetic resonance imaging reconstruction techniques are related to acquisition time reduction and the improvement of image quality. The implementation of these techniques may be the solution for the alleviation of limited scanner availability via workflow acceleration. It can be assumed that this disruptive technology will change daily routines and workflows permanently.

## 1. Introduction

Technical progress and innovative developments have always had an enormous influence on radiology. Whereas the ending of the last century was dominated by the introduction of picture archiving and communication systems, which finally replaced analogous X-ray films, the dominating novelty of this century maybe the implementation of machine learning (ML) and deep learning (DL) architectures and strategies. DL has the potential of being a disruptive technology which may significantly affect radiological workflows and daily clinical practice. 

Convolutional neural networks (CNN) represent one the basic principles of DL algorithms and were already described several decades ago [1]. However, in the very beginning of this work, the success of these algorithms was relatively limited, due to restricted computer processing power. With the introduction of more powerful central processing units (CPU) and graphics processing units (GPU), the success story of DL algorithms truly began [1,2]. Nowadays, studies implementing DL algorithms have even outperformed human readers in certain image recognition tasks [1]. Suitable applications for DL range from lesion or organ detection and segmentation to the classification of lesions [1,3,4,5]. Recent studies also suggest that DL technology can be applied to the prognosis and mortality assessments in cancer and non-cancer patients, opening the perspective for DL-assisted decision support systems [6,7].

However, DL technology has not only been used for the analysis and post-processing of already acquired or reconstructed images, but also for image acquisition and image reconstruction itself, as was shown for both computed tomography (CT) and magnetic resonance imaging (MRI) [8,9,10,11,12,13].

The aim of this article was to present a review of the current DL technologies with a focus on MR image reconstruction. The first part of this article introduces the basic principles of DL image reconstruction techniques. The second part outlines its translation into clinical practice. The third part demonstrates the different fields of applications regarding MR image reconstruction, and different post-processing areas.

## 2. Machine Learning Reconstruction in MRI

Different types of tasks can be solved using machine learning (ML), such as image segmentation, and image classification, as well as regression tasks. While image classification and segmentation assign a global or local label to the input image, MRI reconstruction can be viewed as sensor-to-image translation task. This translation describes an image regression task if continuous predictions are assigned to every pixel in the image.

In MR image reconstruction, we generally aim to recover an image x from the k-space signal y, which is corrupted by measurement noise ϵ, following the equation:

(1) y=Ax+ϵ

where $A\in {\mathbb{C}}^{K\times N}$ is the linear forward operator describing the MR acquisition model, and K denotes the number of measurements, i.e., the dimensionality of the underlying k-space data for the image of N voxels. Depending on the imaging application and signal modelling, the operator A involves Fourier transformations, sampling trajectories, coil sensitivity maps. field inhomogeneities, relaxation effects, motion, and diffusion.

In ML frameworks, the objective is to learn the sensor-to-image mapping function $f_{\Theta }$ with the learnable parameters Θ. The mapping function can be (in the case of deep learning) stated as neural networks and can be used in different ways to reconstruct an image x from the measured k-space data y. All tasks have an image x and/or k-space y as the inputs to the function $f_{\Theta }$, but this can also include further MR-specific information as meta information, e.g., trajectories and coil sensitivity maps. ML reconstruction tasks for MRI differ in terms of input and targeted application output. These reconstruction tasks are further described hereafter.

### 2.1. Image Denoising

Certain types of under-sampled MR acquisition techniques introduce incoherent, noise-like aliasing in the zero-filled reconstructed images. Thus, an image denoising task can be used to reduce the noise-like aliasing in the images. The function $\hat{x}=f_{\Theta }\left(x\right)$ performs an image-to-image regression by predicting the output value $\hat{x}$, which is based on the corrupted input image x. The input to the denoising task can be either the zero-filled (and noise-affected) MR images or the reconstructed MR images that present remaining aliasing or noise amplification for high under-samplings, e.g., images reconstructed with higher parallel imaging acceleration factors. Instead of learning the denoised image, some approaches learn the residual noise that is to be removed from the noisy input [14], as shown for 2D cardiac CINE MRI [15]. The mapping $f_{\Theta }$ only acts on the image x and has no information of the acquired rawdata. Hence, the consistency of the measured k-space signal y cannot be guaranteed. There are approaches that exist which add additional k-space consistency to the cost function [16], or enforce k-space consistency after image denoising, as shown for brain MRIs that have an improvement of image quality [17].

### 2.2. Direct k-Space to Image Mapping

A different ML-based approach is to reconstruct the MR image directly from the acquired k-space data. With the so-called “direct k-space to image mapping”, the k-space data are fed directly into the mapping function to achieve $\hat{x}=f_{\Theta }\left(y\right)$. Consequently, the mapping function approximates the forward model. Learning a direct mapping function is especially useful if the forward model or parts of the forward model are not exactly known. In the case of fully sampled MRIs under ideal conditions, the learned mapping approximates the Fourier transformation [18]. However, this becomes computationally very demanding, due to the fully connected layers which are involved here. Furthermore, the consistency of the acquired k-space data cannot be guaranteed.

### 2.3. Physics-Based Reconstruction

Another family of ML-based MR reconstruction methods is referred to as physics-based reconstruction. These approaches integrate traditional physics-based modelling of the MR which is encoded with ML, ensuring the consistency of the acquired data. We can distinguish two classes of problems here: (1) learning within the k-space domain, and (2) iterative optimization within an image domain containing interleaved data consistency steps. The first approaches are referred to as k-space learning, whereas the latter one is known collectively as unrolled optimization methods. These two approaches can be combined into hybrid approaches that learn a neural network in both the k-space domain and image domain.

### 2.4. k-Space Learning

A prominent approach for physics-based learning in the k-space domain [19] can be viewed as an extension of the linear kernel estimation in the generalized autocalibration partial parallel acquisition (GRAPPA), which is commonly used in parallel imaging acquisitions (e.g., cardiac, musculoskeletal or abdominal MRI). A non-linear kernel which is modelled by the mapping function $f_{\Theta }$ is learned from the autocalibration signal (ACS). The missing k-space lines can then be filled using this estimated, non-linear kernel and the data is then transformed to the image space, using an inverse Fourier transformation. The final image is then obtained by a root-sum-of-squares reconstruction of the individual coil images. The applications of this approach were demonstrated for neuro and cardiac imaging, which showed a superior performance when compared to conventional imaging, especially in the cases of high acceleration factors [19].

### 2.5. Hybrid Learning

Hybrid approaches, as demonstrated in brain MRIs [20], combine the advantages of learning both within the k-space domain and image domain. These networks are applied in an alternating manner to obtain the final reconstruction $\hat{x}$. When designing hybrid approaches, it is important to keep the basic theorems of the Fourier transformation in mind: Local changes in the image domain result in global changes to the k-space domain and vice versa, which should be remembered to avoid unexpected behavior.

### 2.6. Plug-and-Play Priors

Trained image denoisers can also be combined with physics-based learning or conventional iterative reconstructions and serve thus as an advanced regularization for a traditional optimization problem. To achieve this, iterative, image-wise, or patch-wise denoising is performed, followed by a subsequent data consistency step. This concept is also involved in plug-and-play priors [15,21,22,23], regularization by denoising [24], or in image restoration [25], and has been applied in cardiac cine MRI or knee MRI.

### 2.7. Unrolled Optimization

Physics-based learning, which is modelled as an iterative optimization, can be viewed as a generalization of iterative SENSE [26,27] with a learned regularization in the image domain. The basic variational image reconstruction problem

(2) $$\hat{x}\in \arg\underset{x}{\min}\frac{\lambda }{2}\|Ax-y{\|}_{2}^{2}+R\left(x\right)$$

contains a data-consistency term $\|Ax-y{\|}_{2}^{2}$ and a regularization term R(x), which imposes prior knowledge on the reconstruction x. The easiest way to solve Eq. (2) is to use a gradient descent scheme to optimize for the reconstruction x. Alternatively, a proximal gradient scheme [28,29], variable splitting [30] or a primal-dual optimization [31] can be used for algorithm unrolling. In learning algorithms, the iterative optimization scheme is unrolled for a fixed number of iterations to obtain a solution for x. Neural networks replace the gradient of the hand-crafted regularizer R(x) by a learned data-driven mapping function $R\left(x\right)=f_{\Theta }\left(x\right)$. Training several iterations with alternating mapping functions $f_{\Theta }$ and intermittent data consistencies therefore reflect these unrolled optimizations [32]. These networks were shown for a multitude of applications, ranging from neurological [16,33], to cardiac [29,34] to musculoskeletal imaging [35].

**Figure 1:**
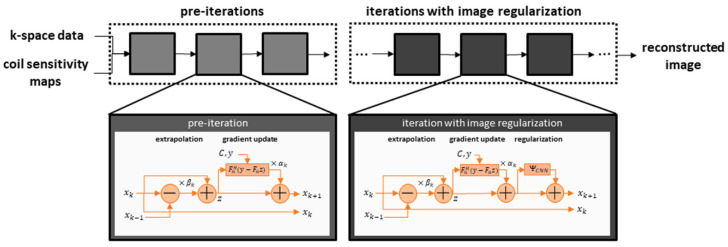
The network receives conventionally determined coil sensitivity maps that specify the local sensitivity of each receiving channel, as well as the under-sampled k-space data. The reconstruction iteratively updates the image based on the gradients of the data fidelity term. In the first step, this may be done without image regularization, as the architecture focuses on generating non-acquired k-space samples which are based on the inherent parallel imaging component of the data fidelity term. As the extrapolation may still involve trainable parameters, such as the gradient step-sizes, these iterations are called pre-cascades. The main deep-learning aspect is then included in subsequent cascades that further include an image-enhancing neural network as regularization.

As our clinical examples shown below also employed networks which relied on unrolled optimization, an exemplary architecture is illustrated in Figure 1.

## 3. Towards Machine Learning Reconstruction in Clinical Practice

While machine learning algorithms have revolutionized multiple fields at an unprecedented speed, and while their limits still need to be explored, the dust is settling for some applications. As data fidelity is essential for medical images, it turns out that the implementation of DL in image reconstructions has to be done very carefully, to avoid, for example, false positives for non-present pathologies, or the hiding of present pathologies. Therefore, a successful strategy for improving image reconstruction is the following: conventional algorithms which consist of multiple processing steps are inspected, and steps that can be replaced by neural networks are identified. The identified steps perform mostly conventional image processing, such as apodization (i.e., optical filtering), interpolation, or denoising. This strategy keeps the conventional modelling for data consistency and parallel imaging untouched, and the introduced neural networks allow for a better tuning of the conventional, often quite simplistic, processing steps. Generally, this reduces the black-box character of the machine learning reconstruction and the requirements of the training data, as well as the mitigation of issues that regard the generalization of applications that were not seen in the training.

An example is given by the development from compressed sensing to variational networks. Compressed sensing relies on a sparsity and incoherence of imaging data, allowing the under-sampling of the k-space [36]. While the reconstructed compressed sensing image can be the solution to an optimization problem, state-of-the-art implementations perform iterative reconstructions that alternate between data consistency and image regularization [37]. While the data consistency is physically motivated and includes a parallel imaging component, the regularization is heuristic. Compressed sensing has introduced the notion of sparsity here, but the justification its use for actual images is rather superficial, and the regularization essentially resembles a form of edge-preserving image denoising. However, in light of machine learning, the typically employed regularizations, which are based on wavelets, can be conceived as small convolutional networks without any trainable components [29,35]. It therefore seems sensible to extend the size of the employed network to allow the better adaptation to MR images and, furthermore, to determine the optimal parameters of a large, representative database through machine learning. From this perspective it is not surprising that the resulting machine learning reconstruction outperforms compressed sensing, and even provides more realistic images. In this context, the specialized term ‘deep learning’ is often used, as the introduced number of trainable parameters in modern architectures is actually larger than may be anticipated. Current architectures often employ well beyond one million trainable parameters and are reported to have a large model capacity. The latter refers to the ability to adapt well to the assigned task of image reconstruction, and this must be supplemented with a suitable amount of training data.

### From the Algorithm to the Scanner—Workflow of Integration

Setting up the architecture for a machine learning reconstruction is the first step. The next step is to decide on a training strategy and to generate training data. If applicable, the most successful strategy is supervised training. In that case, the training data is organized into pairs of input data to be supplied to the machine learning reconstruction, which is then used in the following prospective deployment; the ground truth data is the expected output of the reconstruction. Such training data can be generated through dedicated, long-lasting acquisitions that produce the desired ground truth images that are of a high quality, and the associated input data can be obtained by retrospective under-sampling, i.e., only providing a fraction of the acquired data to the network as an input during training. The training data can be further enhanced through augmentation techniques, such as adding noise, flipping, or mimicking artifacts in the input. It is worth noting that generating training data for machine learning reconstruction seems to have practical advantages when compared to other machine learning tasks, such as segmentation or detection. First, augmentation by a human expert is not necessary, as the target is the already available, high-quality image. Secondly, as the trainable aspect of the network focuses on local image enhancement, such as edges and patterns, the training is less sensitive to the morphological content of the image. The later network performance is mostly determined by a broad range of image contrasts with representative signal-to-noise ratios. For these reasons, the training data can even be acquired on healthy subjects.

The actual training is computationally intensive. It is typically performed on dedicated hardware relying on GPUs, and for actual applications training may last as long as several weeks. For supervised training, a loss function is determined that measures the deviation between the current network output for the provided input data and the ground truth. The trainable parameters of the network are incrementally updated in a process termed backpropagation, which is iterated multiple times while going through the training dataset and which corresponds to an optimization of the trainable parameters of the training dataset. The process is usually tracked by analyzing the network performance on a previously separated validation dataset. This process provides a trained model that can be applied prospectively on data without a known ground truth.

For use in clinical practice, the trained model has finally been deployed on an actual scanner. While the training is typically implemented using freely available software libraries in Python, the hardware limitations of integrated applications often require a translation into a proprietary framework. Here, two aspects are worth mentioning: firstly, the evaluation of a trained network on prospective input data—also called inference or forward pass—is technically easier than the whole training process. Therefore, the requirement for the inference implementation is lower. Secondly, neural networks use a very standardized language to describe their architecture, such as convolutions or activations. This allows the network to perform generic conversions and to not specialize the inference implementation to a specific application. In summary, this strategy allows for the implementation of neural networks that are trained offline into another software environment, e.g., into a routine environment on a clinical scanner.

## 4. Deep Learning Applications in Radiology

As outlined above, DL applications play an important role in radiology, not only for image reconstruction, but also for image classification and segmentation/registration, as well as for prognosis assessment and diagnosis/decision support. Therefore, the applications of DL can be divided into two groups: image acquisition/reconstruction on the scanner and post-processing at the workstation for reporting. Firstly, we would like to highlight image acquisition and reconstruction. 

**Figure 2:**
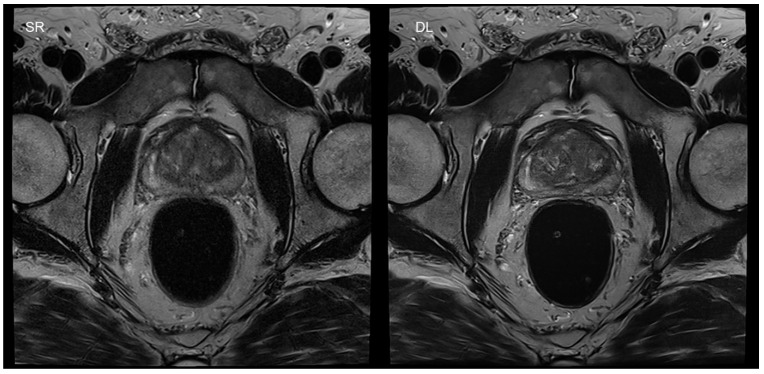
Figure 2 shows an example of a T2-weighted turbo spin-echo (TSE) image of the prostate in the axial plane, with standard reconstruction (SR) and deep learning reconstruction (DL) of a second, under-sampled acquisition of the same patient. SR is shown on the left, with an acquisition time of 4:19 min. The acquisition time of DL was 1:20 min in the same patient (right-hand-side image). Motion artifacts were reduced due to the shortened acquisition time.

An important area for the applications of DL is image reconstruction. It was shown in several studies that a drastic reduction of acquisition time is feasible for clinical applications, using DL-based reconstruction schemes [10,11]. In particular, this approach is of high interest in turbo spin-echo (TSE) imaging. TSE imaging is of utmost importance in musculoskeletal (MSK) and pelvic imaging, due to its robustness and high image quality. However, a severe limitation of these sequences is based on its long acquisition times and reduced scanner availability. In recent studies, it was shown that DL image reconstruction is able to achieve an acquisition time reduction of up to 65% in prostate TSE imaging, without any loss of image quality (Figure 2) [11,38].

**Figure 3:**
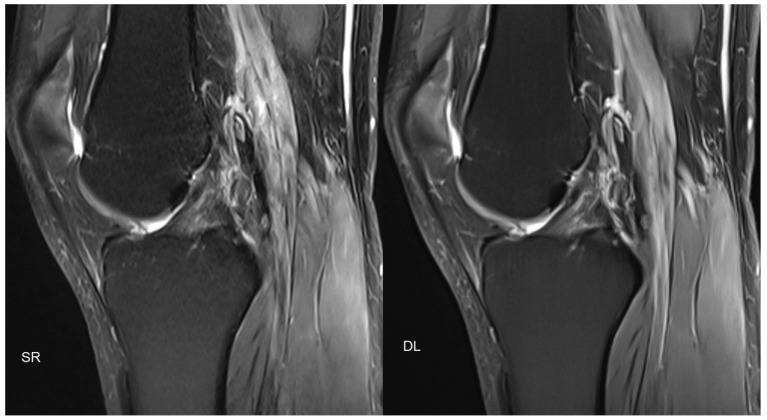
Figure 3 shows an example of PD-weighted turbo spin-echo (TSE) imaging, with fat sat of the knee in the sagittal plane, with standard reconstruction (SR; acquisition time 3:11 min) and deep learning reconstruction (DL; acquisition time 1:33 min) of the same patient. Similar to Figure 2, two different acquisitions were performed (standard acquisition for SR and conventionally under-sampled acquisition for DL).

Similar approaches also exist in MSK imaging, with promising results (Figure 3).

Therefore, DL techniques systematically reducing acquisition times might be the key for overcoming shortness of MR scanner capacities and improve healthcare and patient care. DL image reconstruction can not only be used for reduction of acquisition time, but also for improvement of image quality and improvement of patient comfort as it was presented by Wang et al. in prostate MRI [39]. 

**Figure 4:**
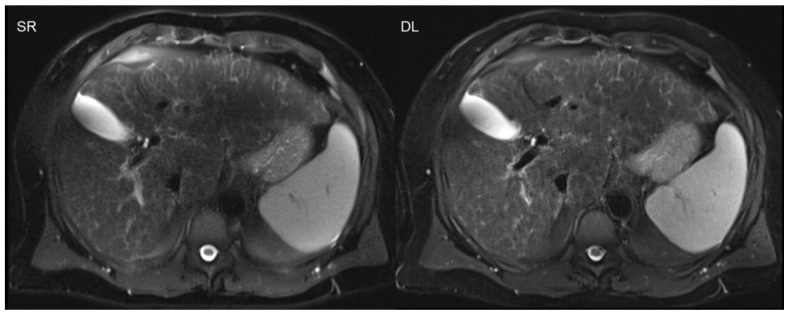
Deep learning reconstruction (DL; acquisition time 0:16 min) of accelerated T2-weighted half-Fourier acquisition single-shot turbo spin-echo sequence (HASTE) of the upper abdomen using a 3T scanner is shown on the right. The left-hand side image demonstrates standard reconstruction (SR; acquisition time 1:30 min).

The advantages of acquisition time reduction are not only limited to time-consuming sequences, as are usually applied in MSK and pelvic imaging. The benefits of scan accelerations are especially advantageous in the imaging of the upper abdomen, e.g., liver imaging, or in cardiac imaging, due to the necessity of breath-holds [34]. Breath-holds often demonstrate challenges in clinical practice regarding motion artifacts, especially in severely ill or elderly patients with restricted breath-holding capacities. The successful implementation of DL was recently also shown in single-shot sequences, such as T2-weighted, half-Fourier acquisition single-shot turbo spin-echo sequences in the upper abdomen (Figure 4) [12,13].

**Figure 5:**
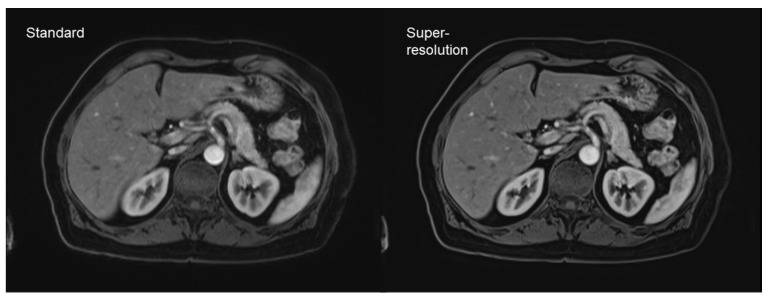
Dynamic contrast-enhanced T1-weighted gradient echo imaging (VIBE Dixon) of the liver. Standard reconstruction is shown on the left. The right hand-side image shows the result of post-processing of the same dataset without change of acquisition parameters, using a deep learning super-resolution algorithm with improved sharpness and contrast.

DL-based super-resolution is another concept worth mentioning. In contrast to simple denoising algorithms, super-resolution aims to increase the spatial resolution via DL-based post-processing [40,41,42]. This concept was successfully implemented in head and neck imaging, as well as abdominal and cardiac imaging [40,43,44]. Especially fast sequences, such as gradient echo (GRE) imaging, benefit from these implementations, due to their relatively low signal-to-noise ratios. The newest algorithm developments also include partial-Fourier imaging with an acquisition time reduction, which is beneficial for the reduction of breathing artifacts (Figure 5) [45].

Due to the increase in radiological examinations and the increase of images that are taken within one examination, additional tools for automated image analysis are very helpful in daily clinical practice. DL networks, especially CNNs, are especially suitable for the further analysis of image data. One of these areas is related to organ segmentation. It was previously shown that the segmentation of the prostate, as well as of the left ventricle, is feasible in MR images using DL networks [46,47]. However, DL networks have not only be applied for segmentation purposes but also for the detection of certain structures and findings. An interesting approach was demonstrated by Dou et al., who applied CNN for the detection of cerebral microbleeds in MRI [48]. It was also previously shown that CNN can be used for the detection and segmentation of brain metastases in MRI [49]. However, the capabilities of novel DL networks even extend to involving classification tasks, in addition to sole detection alone. Wang et al. demonstrated the application of DL in prostate MRI for the classification of prostate cancer [50]. In another study, the impact of DL for the differentiation of clinically significant and indolent prostate cancer was analyzed with promising results using 3T MRI [51]. Furthermore, the potential of DL was also investigated in brain MRI for its ability to distinguish between multiple sclerosis patients and healthy patients [52]. Further developments are related to image registration, as it was demonstrated in the registration of prostate MRI and histopathology images by DL [53].

Besides lesion detection and characterization, it was also demonstrated that DL networks can be applied for the prognosis assessment of patients regarding cancer or non-cancer diseases, e. g. using chest radiographs or MR imaging [6,7]. mpMRI of the prostate, in particular, is of high interest regarding prognosis assessment. It was shown that the prediction of biochemical recurrence after radical prostatectomy via novel support vector machine classification is feasible, with a better performance when compared to standard models [7]. Furthermore, it was demonstrated that machine learning can be helpful for response prediction in intensity-modulated radiation therapy of the prostate using radiomic features prior to irradiation, as well as post-irradiation [54].

## 5. Conclusions

This review outlined state-of-the-art DL-based technologies in radiology, with a focus on image reconstruction in MRI. The promising results of the most recent studies indicate that DL will be a major player in radiology in the upcoming years. Apart from decision and diagnosis support, the major advantages of DL MR reconstruction techniques are related to the acquisition time reduction and the improvement of image quality. Although the future might not yet be present, due to mostly experimental approaches, the next decade will be dominated by DL technologies. The implementation of these techniques may be the solution for the alleviation of limited scanner availability via workflow acceleration. It can be assumed that this disruptive technology will change daily routines and workflows permanently.

## Data Availability

Not applicable.
